# Evolution of Axis Specification Mechanisms in Jawed Vertebrates: Insights from a Chondrichthyan

**DOI:** 10.1371/journal.pone.0000374

**Published:** 2007-04-18

**Authors:** Marion Coolen, Tatjana Sauka-Spengler, Delphine Nicolle, Chantal Le-Mentec, Yvan Lallemand, Corinne Da Silva, Jean-Louis Plouhinec, Benoît Robert, Patrick Wincker, De-Li Shi, Sylvie Mazan

**Affiliations:** 1 Equipe Développement et Evolution des Vertébrés, UMR 6218, Université d'Orléans, Orleans, France; 2 Equipe Développement et Evolution des Vertébrés, UPRES-A 8080, Université Paris-Sud, Orsay, France; 3 Genoscope and UMR Centre National de la Recherche Scientifique (CNRS) 8030, Evry, France; 4 UMR7622, Université Pierre et Marie Curie, Paris, France; 5 Unité de Génétique Moléculaire de la Morphogenèse, URA Centre National de la Recherche Scientifique (CNRS) 2578, Institut Pasteur, Paris, France; Centre de Regulacio Genomica - Barcelona Biomedical Research Park, Spain

## Abstract

The genetic mechanisms that control the establishment of early polarities and their link with embryonic axis specification and patterning seem to substantially diverge across vertebrates. In amphibians and teleosts, the establishment of an early dorso-ventral polarity determines both the site of axis formation and its rostro-caudal orientation. In contrast, amniotes retain a considerable plasticity for their site of axis formation until blastula stages and rely on signals secreted by extraembryonic tissues, which have no clear equivalents in the former, for the establishment of their rostro-caudal pattern. The rationale for these differences remains unknown. Through detailed expression analyses of key development genes in a chondrichthyan, the dogfish *Scyliorhinus canicula*, we have reconstructed the ancestral pattern of axis specification in jawed vertebrates. We show that the dogfish displays compelling similarities with amniotes at blastula and early gastrula stages, including the presence of clear homologs of the hypoblast and extraembryonic ectoderm. In the ancestral state, these territories are specified at opposite poles of an early axis of bilateral symmetry, homologous to the dorso-ventral axis of amphibians or teleosts, and aligned with the later forming embryonic axis, from head to tail. Comparisons with amniotes suggest that a dorsal expansion of extraembryonic ectoderm, resulting in an apparently radial symmetry at late blastula stages, has taken place in their lineage. The synthesis of these results with those of functional analyses in model organisms supports an evolutionary link between the dorso-ventral polarity of amphibians and teleosts and the embryonic-extraembryonic organisation of amniotes. It leads to a general model of axis specification in gnathostomes, which provides a comparative framework for a reassessment of conservations both among vertebrates and with more distant metazoans.

## Introduction

It is currently clear that the genetic control of development in metazoans relies on a limited number of signalling pathways and broad families of transcription factors, organised into at least partially conserved regulatory networks, which have been repetitively reused and reshaped during evolution in different cellular contexts [1]. The discovery of these conservations had a major impact on the rise of modern embryology, by opening the possibility to transpose the results obtained in a given model organism, such as drosophila, to a much broader range of species, for instance vertebrates. Furthermore, comparisons between distantly related model organisms have shown that even more unexpectedly, similarities are not restricted to the regulatory modules themselves but extend to the developmental processes or the cellular phenotypes that they control [2], [3]. However, the conservation of the complex sequences of cellular and genetic interactions controlling developmental processes can be difficult to assess, even among relatively closely related species. Different reasons account for this limitation. First, the underlying conservation of developmental processes can be obscured by the use of different experimental approaches, highlighting different aspects of the same phenomena, or by the presence of paralogous genes, resulting in function shuffling [4], [5]. In the absence of systematic comparative analyses, the gaps in our knowledge of the regulatory networks and successive inductive events that control developmental processes also considerably complicate comparisons between model organisms, certain aspects being particularly obvious in one species but less readily recognisable in another one [6]. Finally, the accumulation during evolution of species- or taxa-specific morphological features, resulting themselves from changes at the molecular level, can make it extremely difficult to define homologies between cell populations and thus establish the link between model organisms on robust bases [7].

The mechanisms which underlie early axis specification in vertebrates provide an illustration of such difficulties. Our current understanding of this process, which mainly relies on studies conducted in the chick, mouse, xenopus and zebrafish, suggests highly divergent mechanisms between amniotes, amphibians and teleosts. The latter two share a number of important characteristics. Both display an early dorso-ventral polarity, which is established upon fertilisation by the relocalisation of maternal determinants towards the dorsal side of the embryo. This results in a complex cascade of inductive events and genetic interactions between the dorsal and ventral sides, which leads to the formation of the organizer and the three germ layers at the onset of gastrulation [reviewed in 8], [9]. The conservation of these early genetic interactions in amniotes remains an opened question. Until recently, it was generally considered that radial symmetry was only broken at relatively late, pre-gastrulation stages in the chick and mouse, by a coexpression of *Vg1* and *Wnt8* at the level of the posterior marginal zone (PMZ) in the former [10], [11] and by the polarised, anterior displacement of an extraembryonic cell population, the anterior visceral endoderm (AVE), in the latter [12], [13]. This view has recently been challenged in the mouse and it is currently clear that the embryo is endowed with a bilateral symmetry as early as the blastocyst stage [14]–[17]. However, the relationship of this early polarity to the position and antero-posterior orientation of the embryonic axis remains an open question. Furthermore, at least in the chick, this polarity retains a considerable plasticity until pre-gastrulation stages, as shown by experimental manipulations of the blastoderm [18], [19], [reviewed in 20]. A further complication for a unifying view of the mechanisms of axis formation in vertebrates comes from the observation that in amniotes, extraembryonic tissues (epiblast but also trophectoderm derivatives in the mouse), play an important part in early antero-posterior patterning [21]–[26]. These tissues have no clear homologues in zebrafish and xenopus, even though candidate AVE equivalents have been proposed [27]–[32].

In order to better understand the link between amniotes, amphibians and teleosts, we have used an evo-devo approach, aimed at the reconstruction of the ancestral molecular mechanisms controlling early axis specification in jawed vertebrates. The rationale for this approach is based on the fact that all species are linked by common descent from the ancestral gnathostome state. A better understanding of this state can therefore help to identify species- or taxa-specific features, which obscure the relationships between current model organisms. In this approach, we chose to focus on a chondrichthyan, the dogfish *Scyliorhinus canicula*. Three main reasons underlay this choice. First, as the sister group of osteichthyans, chondrichthyans provide the closest outgroup to amniotes, amphibians and actinopterygians. This phylogenetic position allows reliable morphological comparisons with vertebrate model organisms, which is important to identify gnathostome primitive characteristics and is less obvious with more distant chordates, such as ascidians or cephalochordates. Similarly, at the molecular level, relatively low sequence divergence rates and the presence of shared orthology classes allow robust phylogenetic reconstructions and the unambiguous identification of orthologues of genes characterised in vertebrate model organisms [33]. Second, embryonic series can be relatively easily obtained starting from early stages and development tables have been established in this species [34]. Finally, the dogfish embryo develops as a broad flat blastoderm lying on a large mass of undivided yolk and displays expanded, distinct, extraembryonic tissues, which can facilitate comparisons with amniotes [34], [35]. The detailed histological and molecular characterization of the dogfish early embryo highlights striking similarities not only with amphibians and teleosts but also with amniotes. Taken together, our data suggest an unexpectedly conserved molecular pattern, likely to reflect the ancestral state of jawed vertebrates, and lead to a model, which provides a basis for a synthetic view of early antero-posterior patterning mechanisms among vertebrates.

## Results

### Histological characterization of the dogfish embryo at pre-gastrulation and gastrulation stages

**Figure 1:**
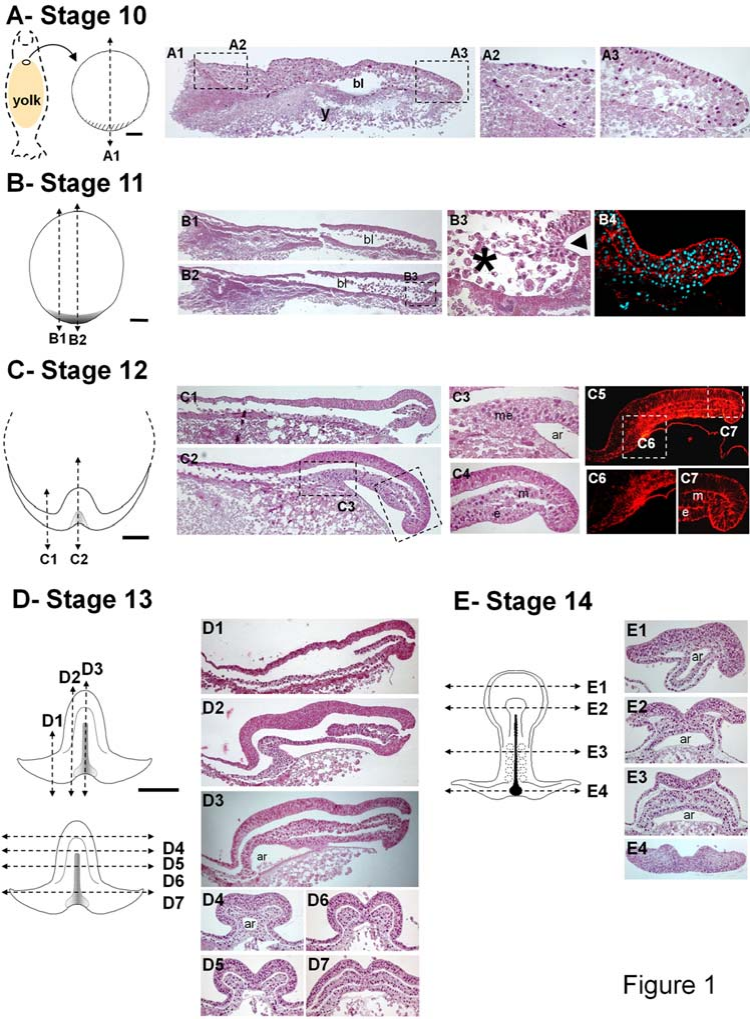
Histological sections of dogfish embryos from stages 10 to 14. For each stage, a schematic animal view of the embryo is shown on the left, anterior to the top. Dotted lines on this view indicate the level and plane of the sections shown. Sagittal sections are shown with anterior to the left and posterior to the right. Sections A2–A3, B3, C3–C4, C6–C7 are respectively higher magnifications of sections A1, B1, B2, C2, C5. Sections A1–A3, B1–B3, C1–C4, D1–D7, E1–E4 are stained with hematoxylin/eosin. Section B4 shows a high magnification of central posterior margin stained with rhodamine-phalloidin and DAPI. Section C5 is stained with phalloidin. In B3 the arrowhead points to bottle-like cells and the asterix shows the location of elongated cells close to the posterior margin. Scale bar: 500 µm. Abbreviations: ar, archenteron; bl, blastocoele; e, endoderm; m, mesoderm; me, mesendoderm; y, yolk.

We have previously reported the main morphological characteristics of the dogfish prior and during gastrulation [36]. In order to obtain a detailed histological characterization, we performed serial sections of embryos starting from stage 10, which precedes the onset of gastrulation, to stage 14, characterized by the individualisation of the anterior-most somites (Fig. 1). As previously reported [36], the appearance of a thickening at the posterior margin of the blastoderm is a hallmark of stage 10 (Fig. 1A1,A3). At this stage, the blastoderm consists of a simple cuboidal epithelium overlying a mesenchymal population of round shaped cells (Fig. 1A1–A3). Anterior to the blastocoel, this mesenchyme forms a dense cell population (Fig. 1A2), whereas posteriorly cells appear more dispersed (Fig. 1A3). At stage 11, the cuboidal epithelium persists in the anterior moiety of the blastoderm and spreads by epiboly over the yolk, concomitantly with a marked thinning of the underlying mesenchymal cell population (Fig. 1B1,B2). In contrast, the histological structure of the blastoderm substantially changes in its posterior half. At this level, the superficial epithelial layer becomes pseudostratified and folds inward over a 60° sector of the posterior margin (Fig. 1B3,B4). It thus forms an overhang, which overlays the forming anterior part of the archenteron. In the anterior part of the involuted cell layer, epithelial cells now display a pyramidal shape, reminiscent of the xenopus bottle cell population (Fig. 1B3). An additional population of dispersed elongated cells becomes visible adjacent to the former. These cells are devoid of cortical phalloidin staining, consistent with a change of the cytoskeleton structure (Fig. 1B4). Together with the characteristic anterior to posterior orientation of their processes (Fig. 1B3), this is suggestive of an epithelial to mesenchyme transition occuring at this level. Stage 12 is characterized by the first clear appearance of the embryonic axis and the segregation of the three germ layers at the posterior margin (Fig. 1C1,C2). Distinct mesoderm and endoderm layers are visible next to the margin (Fig. 1C4), whereas in the anterior midline, a single mesendoderm layer overlies the anterior tip of the archenteron (Fig. 1C3). This mesendoderm layer is continuous with the loose mesenchymal population of elongated cells, which extends over the yolk membrane (Fig. 1C2). At this stage, the neural plate becomes distinct from the adjacent surface ectoderm and the neural folds elevate. These broad features persist at later stages (Fig. 1D,E), which are characterized by the extension of the embryonic axis and the individualisation of the first somites (stage 14). Both anteriorly and laterally, the embryonic ectoderm and mesendoderm are continuous with presumably extraembryonic outer epithelial and inner mesenchymal layers, spreading over the yolk.

### Regionalisation of the posterior arms during embryonic axis extension

**Figure 2:**
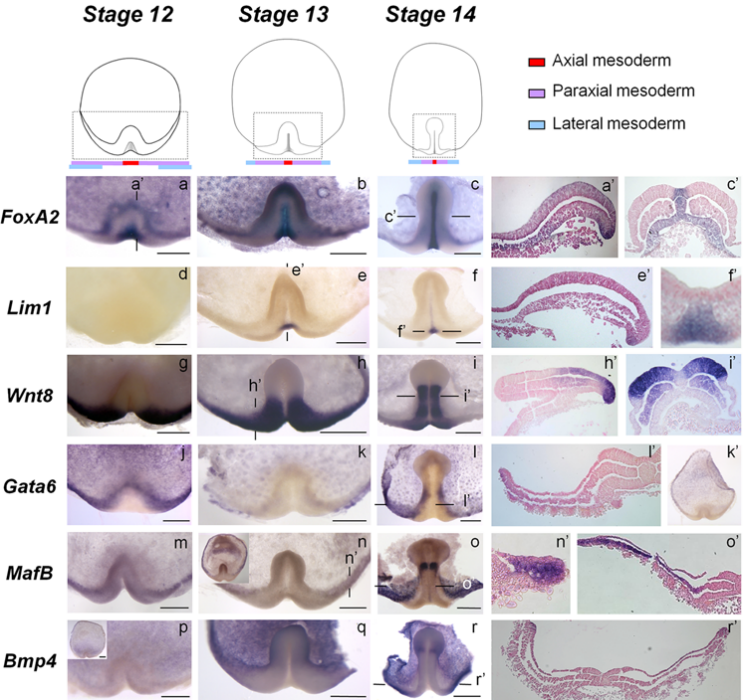
Expression of mesoderm regional markers during axis extension. Animal views of *S. canicula* embryos after whole-mount in situ hybridization using *FoxA2* (a–c), *Lim1* (d–f), *Wnt8* (g–i), *Gata6* (j–l, k'), *MafB* (m–o) and *Bmp4* (p–r) probes. Views are restricted to the territories enclosed in a dotted box on the schemes of the upper panel. In this panel, below the schematic views of embryos, colored bars symbolise the largely exclusive, more and more lateral, expression territories of markers of axial (red), paraxial (purple) and lateral (blue) mesoderm at the posterior margin. A', c', e', f', h', i', l', n', o', r': sections of the embryos shown in a, c, e, f, h, i, l, n, o, r after eosin counterstaining. The planes of sections are indicated by thin lines on the whole-mount view of each embryo. Scale bar: 500 µm.

Analysis of *Brachyury* expression in *S. canicula* had led us to point to the posterior margin as the major site of mesoderm internalisation during gastrulation [36]. To further address this hypothesis, we analysed expression of molecular markers of axial, paraxial and lateral mesoderm during gastrulation (stages 12 to 14). As in osteichthyans [37]–[41], dogfish *FoxA2, Wnt8,* *Gata6* and *MafB* are expressed in different mesodermal components of the newly formed embryonic axis (stage 14). *FoxA2* expression is detected in all midline tissues, as well as the whole forming gut (Fig. 2c,c'). *Wnt8* expression territory lies lateral to the midline *FoxA2* positive tissues, both in the paraxial mesoderm and neuroectoderm, excluding the cephalic enlargement (Fig. 2i, i'). *Gata6* shares with its osteichthyan orthologs [42]–[44] a specific expression in the lateral mesoderm, at the precardiac level and posterior to it (Fig. 2l, l'). Finally, *MafB* shares with *Gata6* an expression in the lateral mesoderm but also extends to the intermediate mesoderm (Fig. 2o, o'). In all cases, these territories within the forming embryonic axis are continuous with specific expression domains along the posterior margin. At this level, the medial to lateral extent of these domains differs between the markers studied, reflecting their axial to lateral distribution in the newly formed mesoderm. At stage 14, the marginal expression of *FoxA2* is thus restricted to the notochordal triangle (Fig. 2c), where it appears largely overlapping with the expression territory of another organizer marker, *Lim1* (Fig. 2f, f') [45]–[47]. At this stage, *Wnt8* expression domain lies on each side of this territory, in the bulging posterior arms (Fig. 2i). *MafB* and *Gata6* transcripts are largely excluded from this presumptive paraxial mesoderm territory and are restricted to more lateral parts of the posterior margin (Fig. 2l, o). This regionalisation of the posterior margin is already observed at earlier stages, from stage 12 to 13 (Fig. 2a, b, e, g, h, j, k, m, n). We also note that expression of *MafB* extends laterally along the margin, including its anterior-most aspect (Fig. 2n). Similarly *Bmp4*, another marker of ventral mesoderm in amphibians [48], can be detected along the anterior and lateral parts of the margin (Fig. 2p, q, r, r'), suggesting that an additional minor mesoderm cell population may also be internalised at these levels.

### A temporal regulation of embryonic axis formation from rostral to caudal levels

**Figure 3:**
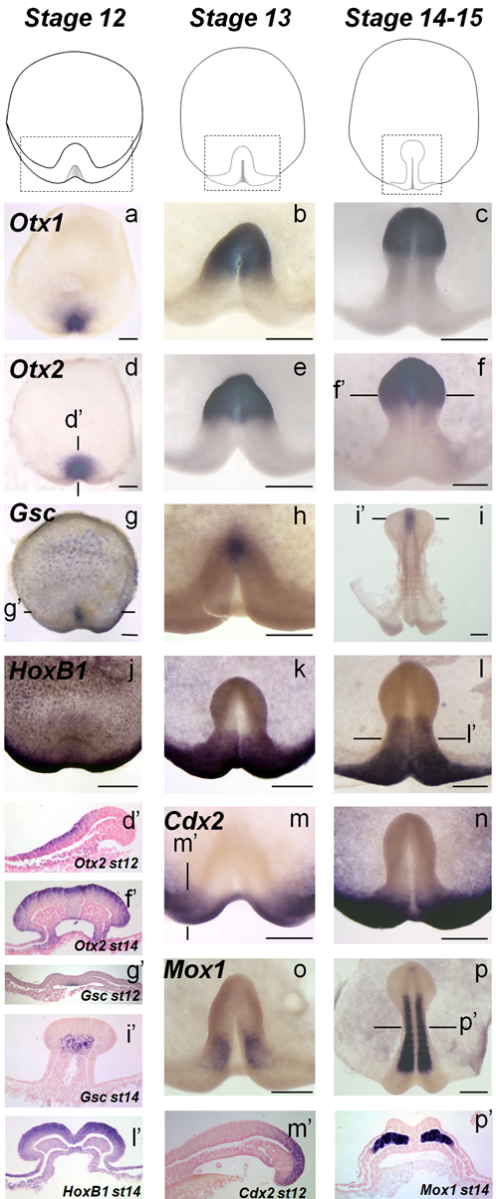
Temporal regulation of embryonic axis formation from rostral to caudal level. Animal views of *S. canicula* embryos after in situ hybridization using *Otx1* (a–c), *Otx2* (d–f), *Gsc* (g–i), *HoxB1* (j–l), *Cdx2* (m,n) and *Mox1* (o, p) probes. For each probe, stages are indicated in the upper line. The views are focussed on the territories enclosed in dotted boxes in b, c, e, f, h, i, j, k, l, m, n, o, p. d', f', g', i', l', m', p': sections of hybridized embryos shown in d, f, g, i, l, m, p after eosin counterstaining. The planes of sections are indicated by thin lines on the whole-mount view of each embryo. Scale bar: 500 µm.

In order to better understand embryonic axis formation in the dogfish, we next analysed the relative timing of expression of molecular markers expressed at different antero-posterior levels in the elongating embryonic axis, starting from its morphological appearance (stage 12) to somite stages (stages 14–15). In all cases studied, the onset of expression of the rostral markers studied (*Otx1*, *Otx2*, *Gsc*) preceded the one of more caudal markers (*HoxB1*, *Cdx2*, *Mox1*). At stages 13 to 14, *Otx1* and *Otx2* expression territories largely overlap in the anterior neural plate, with a sharp posterior boundary, later coincident with the mesenphalon-metencephalon border (Fig. 3b, c, e, f, f'). This characteristic expression domain, which has been observed in all vertebrate species studied [49]–[53], is already established by stage 12 in a discrete cell population of the upper prospective neuroectoderm layer, about 10 cell diameters far from the posterior margin (Fig. 3a, d, d'). Similarly, in the dogfish, *Gsc* shares with its osteichthyan counterparts [54]–[57] a highly specific expression territory in the prechordal plate, which can be unambiguously identified starting from stage 13 (Fig. 3h, i, i'). This *Gsc* expression territory is already apparent at stage 12 in the mesendoderm cell layer underlying the *Otx* positive territory (Fig. 3g, g'), suggesting that specification of the prospective forebrain and midbrain neuroectoderm and the underlying mesendoderm has taken place. In contrast, none of the trunk molecular markers studied can be detected in the newly formed embryonic axis at this stage. *HoxB1* transcripts are restricted to a 90° crescent shaped sector of the posterior margin (Fig. 3j) and only appear in the adjacent posterior part of the elongating embryonic axis starting from stage 13, with the same transcript distribution as in osteichthyans (prospective hindbrain, paraxial mesoderm [58], [59]) (Fig. 3k, l, l'). Similarly, as in osteichthyans [60]–[62], *Cdx2* et *Mox1* expressions are confined to the posterior-most part of the forming embryonic axis (neuroectodermal and endodermal layers for the former; paraxial mesoderm for the latter) (Fig. 3m, n, o, p, m', p'). In both cases, expression is first detectable at stage 13. These data suggest that, as in osteichthyans, the embryonic axis also elongates following a rostral to caudal progression in the dogfish. This process may be subjected to a particularly tight temporal control in this species. We thus observe that the three germ layers are fully established at a very short distance from the posterior margin where mesendoderm internalisation takes place.

### Expression of hypoblast and organizer markers at the posterior margin of the blastoderm at pre-gastrulation stages

**Figure 4:**
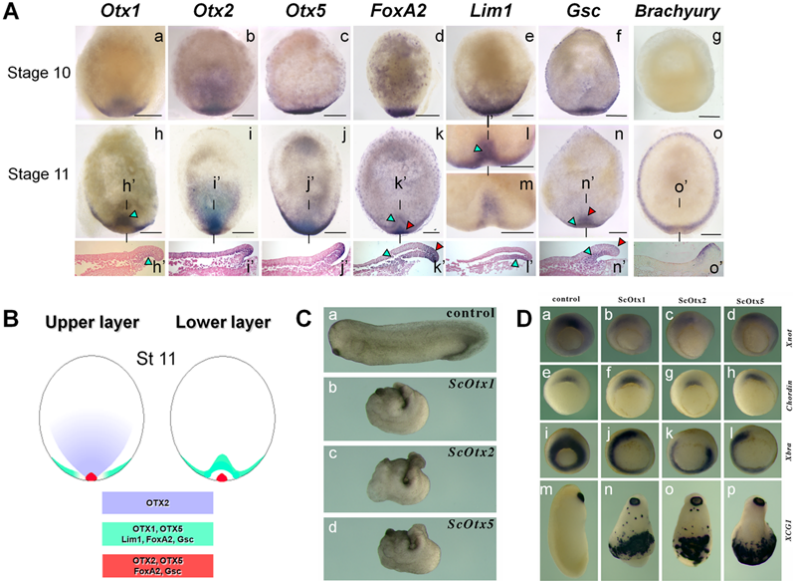
Molecular characterization of the dogfish embryo at pre-gastrula stages. A) Animal views of *S. canicula* embryos after in situ hybridization using *Otx1* (a,h), *Otx2* (b,i), *Otx5* (c,j), *FoxA2* (d,k), *Lim1* (e,l,m), *Gsc* (f, n) and *Brachyury* (g, o) probes at stage 10 (upper line) and 11 (second line) as indicated. h', i', j', k', l', n' and o' are midline sagittal sections through the embryos shown in h, i, j, k, l, n and o respectively. Scale bar: 500 µm. B) Summary of the three main expression territories identified at stage 11 on the basis of gene expression patterns. Left panel: transcript distribution in the upper cell layer of the blastoderm. Right panel: transcript distribution in the lower layer of the posterior overhang. C) Phenotypes of *Xenopus* embryos injected with dogfish *Otx* mRNAs. Embryos at four-cell stage were dorsally-injected with 100 pg dogfish *Otx* mRNA. They were cultured to stage 35 for score of phenotypes. (a) An uninjected embryo. (b) A dogfish *Otx1*-injected embryo. (c) A dogfish *Otx2*-injected embryo. (d) A dogfish *Otx5*-injected embryo. Overexpression of all three dogfish Otx proteins leads to very similar phenotypes. D) Expression of mesoderm and cement gland markers in dogfsih *Otx*-injected *Xenopus* embryos. The embryos shown in the second, third and fourth column were respectively injected with 100 pg of dogfish *Otx1*, *Otx2* and *Otx5* mRNA, controls are shown in the first column. The embryos hybridized with *Xnot2* (first line), *chordin* (second line) and *Xbra* (third line) were injected in the dorsal region at the four-cell stage and developed until stage 11, those hybridized with the *XCG* probe (fourth line) were injected in the ventral region and developed until stage 25. (a) Control embryo showing *Xnot2* expression in the dorsal mesoderm. (b, c, d) Injection of dogfish *Otx1* (b), *Otx2* (c) and *Otx5* (d) inhibits *Xnot2* expression. (e) Control embryo showing chordin expression. (f, g, h) Injection of dogfish *Otx1* (f), *Otx2* (g) and *Otx5* (h) did not affect chordin expression. (i) Control embryo showing Xbra expression in the entire maginal mesoderm. (j, k, l) Injection of dogfish *Otx1* (j), *Otx2* (k) and *Otx5* (l) inhibits *Xbra* expression at the sites of injection. (m) Control stage 25 embryo showing XCG1 expression in the cement gland. (n, o, p) Injection of dogfish *Otx1* (n), *Otx2* (o) and *Otx5* (p) strongly induced ectopic XCG1 expression in the ventral region.

In amniotes, head formation requires secreted signals from two signalling centers, the hypoblast (or anterior visceral endoderm in the mouse) and the early gastrula organizer, also characterized in all vertebrate model organisms. These two cell populations share a set of specific genetic markers including *Lim1*, *FoxA2*, *Gsc* and *Otx2* homeodomain genes [reviewed in 63]. In order to identify possible homologous territories in the dogfish embryo, we analysed expression of these markers, including the three dogfish *Otx* paralogues. We performed this analysis at stages 10 and 11, which precede head formation. At stage 10, the five genetic markers display a very similar expression territory spanning a broad crescent shaped sector of the posterior margin (Fig. 4A a–f). Their expression patterns segregate at stage 11 (Fig. 4A h–n), when *Brachyury* expression becomes first detectable in a ring-shaped ectodermal domain abutting but excluding the blastoderm margin (Fig. 4A o, o'). At this stage, all, with the notable exception of *Otx2*, share an expression territory in the lower layer (Fig. 4A h'–n'). The signals obtained with these markers are not completely superimposable but all exhibit the same dynamics. They are characterized by a convergence, along the margin and towards the posterior midline, of the crescent shaped signal previously observed, and a subsequent anterior displacement of the labeling in the lower layer, at a distance from the posterior margin. A second labelled territory, shared by *Otx2*, *Otx5* and *FoxA2* (Fig. 4A i'–k'), persists at the margin, concentrated at the midline at the location where the notochordal triangle later forms. This expression site is continuous with the first territory in the case of *Otx5* (Fig. 4A j') but clearly distinct in the case of *FoxA2* (Fig. 4A k'). *Lim1* and *Gsc* are also expressed at this location but with different characteristics. The *Lim1* notochordal triangle expression is only detected in a later phase, starting from stage 13 (Fig. 2e), while the second *Gsc* expression site is initiated in the upper epiblast layer (Fig. 4A n'), transiently observed at the margin and later found in the prospective prechordal plate as described above (Fig. 3g–i). Taken together, these data highlight the presence at stage 11 of two distinct territories respectively located in the lower layer and at the posterior margin, showing largely overlapping *Lim1*, *Gsc* and *FoxA2* signals and expressing different combinations of *Otx* paralogues (Fig. 4B). In addition, *Otx2* displays a widespread epiblast expression domain that is unique to this paralogue. This domain extends to the central part of the blastoderm but excludes its lateral parts as well as the anterior-most cuboidal epithelium (Fig. 4A b, i).

A single *Otx* paralogue, *Otx2*, is expressed during and prior to mouse gastrulation [64]. The expression patterns observed in the dogfish suggest that in contrast, early functions may be partitioned between the three paralogs in this species. In order to test whether dogfish paralogous proteins are endowed different biochemical properties, we used microinjections of synthetic dogfish *Otx1*, *Otx2* and *Otx5* mRNAs in xenopus embryos. No indication of such differences could be obtained using this experimental test. Dorsal injections of the three paralogous forms at the four-cell stage resulted in identical phenotypes, characterized by posterior truncations (Fig. 4C) in all injected embryos (Table 1).To further study the effects of the overexpression, we assayed in injected embryos the expression of *Xnot2*, *Xbra* and *chordin*, which are respectively tailbud, presumptive mesoderm and organizer markers (Fig. 4D). The expression of *Xnot2* was absent or strongly reduced in 9 out of 10 embryos injected with dogfish *Otx1* and *Otx2* mRNA, and in 6 out of 10 embryos injected with dogfish *Otx5* mRNA, which provides an indication of posterior truncations in all three cases. More than 90% of the embryos injected with dogfish *Otx1* (n = 20), *Otx2* (n = 20) or *Otx5* (n = 21) mRNAs showed local repressions of *Brachyury* expression along the marginal zone. In contrast, *chordin* expression was unaffected in all embryos tested (n = 20 for all three paralogs). These results strongly suggest that overexpression of all three paralogs induces posterior truncations, related to inhibition of convergence-extension movements. Similarly, ventral injections led in all cases to ectopic inductions of anterior structures, as assessed by expression of the cement gland marker *XCG* (Fig. 4D m–p). These phenotypes are identical to those previously reported upon overexpression of osteichthyan Otx proteins [65], suggesting a functional equivalence of the three dogfish Otx proteins.

**Table 1:** Effect of microinjecting dogfish *Otx* mRNAs in *Xenopus* embryos.

	Phenotypes			
mRNA	Normal	Dorsal defects	Trunk/Posterior truncations	*n*
Uninjected	100			45
ScOtx1		96	100	27
ScOtx2		100	100	37
ScOtx5		97	100	37

Phenotypes of injected embryos were scored at neurula ans late tail-bud stages. Dorsal defects indicate failure of neural tube closure and open blastopore at neurula stage, and trunk and posterior truncation refers to embryos with reduced trunk and tail structures at late tail-bud stage. The results are expressed as percentages, except n, which refers to total injected embryos.

### Highly dynamic expression patterns of signalling molecules prior to axis formation

**Figure 5:**
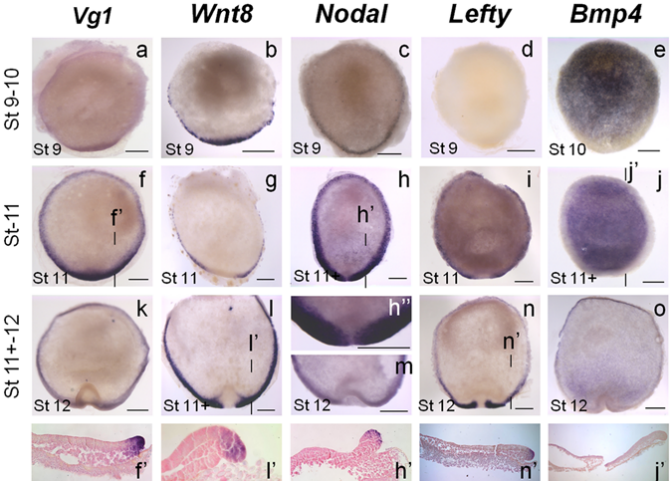
Expression profiles of signalling molecules in the dogfish from pre- to early gastrulation stages. a–n: animal views of dogfish embryos hybridized with *Vg1* (a, f, k), *Wnt8* (b, g, l), *Nodal* (c, h, m), *Lefty* (d, i, n), *Bmp4* (e, j, o) probes. f', l', h', n', j' are sagittal sections through the embryos shown in f, l, h, n, j respectively, after eosin counterstaining. The planes of sections are indicated by thin lines on the whole-mount view of each embryo. h” is a higher magnification of h at the level of the posterior margin. Similarly the view in m is restricted to the posterior margin. Scale bar: 500 µm.

In order to further address signalling centers of the dogfish embryo, we next analysed the transcript distribution of five secreted molecules known to be involved in axis specification in osteichthyans: Vg1, Wnt8, Nodal, Bmp4 and Lefty. This analysis was carried out at early stages of axis formation (stages 9 to 12). All five markers exhibit highly specific expression territories at these stages. *Vg1*, *Wnt8*, *Nodal* territories overlap at the posterior margin at stage 9 (Fig. 5a, b, c) and segregate at subsequent stages. At stage 11, *Wnt8* expression domain spans broad posterior and lateral sectors of the margin but excludes its medial part, positive for organizer and hypoblast markers (Fig. 5g). The signal then becomes confined to the forming posterior arms at stage 12 as described above (Fig. 5l, l'). *Vg1* expression at stage 11 is very different, spanning the whole periphery of the blastoderm with the highest signal intensity at the posterior margin (Fig. 5f, f'). This peripheral expression persists at stage 12, albeit with a lower signal intensity (Fig. 5k). *Nodal* territory, which is restricted to a narrow row of cells at stage 9 (Fig. 5c), markedly expands to form a broad marginal ring at stage 11 (Fig. 5h, h'). A gradient of signal intensity is observed from anterior to posterior, the transcripts being nevertheless excluded from the posterior midline with sharp boundaries (Fig. 5 h”). This expression territory persists at later stages, albeit with a significantly lower signal intensity (Fig. 5m). *Lefty* expression, which is first observed at stage 11 (Fig. 5i, n, n'), largely parallels that of *Nodal* along the margin. *Bmp4* expression pattern substantially differs from the formers. At stage 10, it spans a broad blastoderm area excluding a posterior crescent-shaped territory with diffuse posterior boundaries (Fig. 5e). These boundaries markedly sharpen at stage 11 (Fig. 5j, j'). At this stage, *Bmp4* and *Nodal* expression territories appear largely complementary. At stage 12, *Bmp4* persists at lower levels in the upper epiblast layer, but is excluded from the forming neural plate and posterior arms. It also becomes detectable along the anterior blastoderm margin excluding the posterior arms (Fig. 5o; see also Fig. 2p).

### An early bilateral axis defined by opposite *Bmp4* and *Otx5/Nodal* territories starting from blastula stages

**Figure 6:**
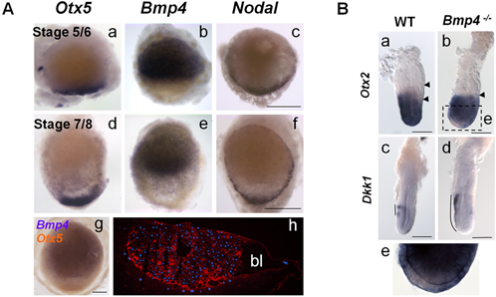
Early polarities at blastula stages in the dogfish. A. a–f: animal views of early dogfish embryos hybridized with *Otx5* (a,d) , *Bmp4* (b, e) and *Nodal* (c, f) probes. g: double in situ hybridization using *Bmp4* (blue) and *Otx5* (brown) probes at stage 9. h: sagittal section through a stage 7/8 dogfish embryo stained with rhodamine-phalloidin (red) and DAPI (blue). At this stage, the embryo consists in a mass of round-shaped cells lying on top of the vitellus. A single layer of elongated cells is visible at the level of the blastocoele roof. Scale bar: 500 µm. B. Abnormal expression of anterior markers in *Bmp4* null mutant embryos. Lateral views of wild-type (WT: a, c) and Bmp4^tm1Blh^/Bmp4^tm1Blh^ (*Bmp4^-/-^* :b, d) 6.5 dpc mouse embryos, hybridized with *Otx2* (a, b) and *Dkk1* (c, d) probes. In *Bmp4^-/-^* embryos, *Otx2* transcripts are not restricted anteriorly and a distal extension of the *Dkk1* territory in the anterior visceral endoderm is visible. e: Higher magnification of the embryo in e, showing the absence of *Otx2* transcripts in the distal part. Scale bar: 100 µm.

The molecular characterization of the dogfish embryo shows that a clear molecular asymmetry of the blastoderm, which reflects the later antero-posterior polarity of the embryo proper, is established at pre-gastrula stages. In order to address when this asymmetry is first established, we extended our molecular characterization of the dogfish embryo to stages preceding blastocoele formation (stage 5/6) or slightly following it (Stage 7/8) (Fig. 6A h). Expression remained undetectable for the majority of the markers described above, except for three of them, *Bmp4*, *Nodal* and *Otx5*. At the two stages studied, *Nodal* and *Otx5* display prominent and highly specific signals restricted to a discrete cell population lying at the posterior border of the blastoderm, adjacent to the blastocoele when it becomes visible (Fig. 6A a, c, d, f). *Bmp4* expressing domain encompasses a broad blastoderm sector (Fig. 6A b, e), lying opposite to the *Otx5* and *Nodal* positive posterior margin (Fig. 6A g). In the dogfish, *Bmp4*, *Nodal* and *Otx5* expression territories thus define an early polarity, which precisely reflects the later antero-posterior polarity of the embryonic axis.

This striking feature of dogfish embryos prompted us to test a possible involvement of *Bmp4* in antero-posterior patterning in osteichthyans, using *Bmp4-/-* mouse embryos. At the onset of gastrulation, *Otx2* transcripts, which are initially present in the whole epiblast, normally regress to the prospective anterior side of the embryo and withdraw from the proximal posterior region where the primitive streak forms (Fig. 6B a). This anterior displacement of the labelling was not observed in any of the *Bmp4-/-* embryos analysed (3/3) (Fig. 6B b). In addition, all these embryos displayed an absence of *Otx2* signal in a discrete distal cell population of the visceral endoderm (Fig. 6B e). We next analysed expression of *Dkk1*, which is a Wnt antagonist selectively expressed in the AVE (Fig. 6B c). All *Bmp4-/-* embryos (2/2) showed the expected anteriorisation of *Dkk1* signal at 6.5 dpc (Fig. 6B d). However, a significant distal expansion of the transcripts was also observed in all cases. These data remain prelimary and will have to be extended using a wide range of genetic markers. However they show that predictions inferred from an unexpected characteristic of the dogfish can be used to address novel hypotheses in a model organism.

## Discussion

In the absence of developmental genetics and experimental embryology techniques, addressing the mechanisms which control early development remains difficult in the dogfish. Considering these limitations, an alternative approach is to study the dynamic of gene expression patterns at blastula and early gastrula stages.This approach does not provide insight into the conservation of mechanisms proper but allows comparisons of the successive specification states that predate axis formation. In the present study, the dogfish early embryo turned out to be remarkably suited to such a comparative approach, due to an excellent spatial and temporal resolution of the molecular profiles observed. Comparisons of this highly dynamic molecular pattern with vertebrate model organisms highlight unexpected similarities and provide new insights into the gnathostome ancestral state.

### Similar modes of gastrulation in chondrichthyans and in amniotes

**Figure 7:**
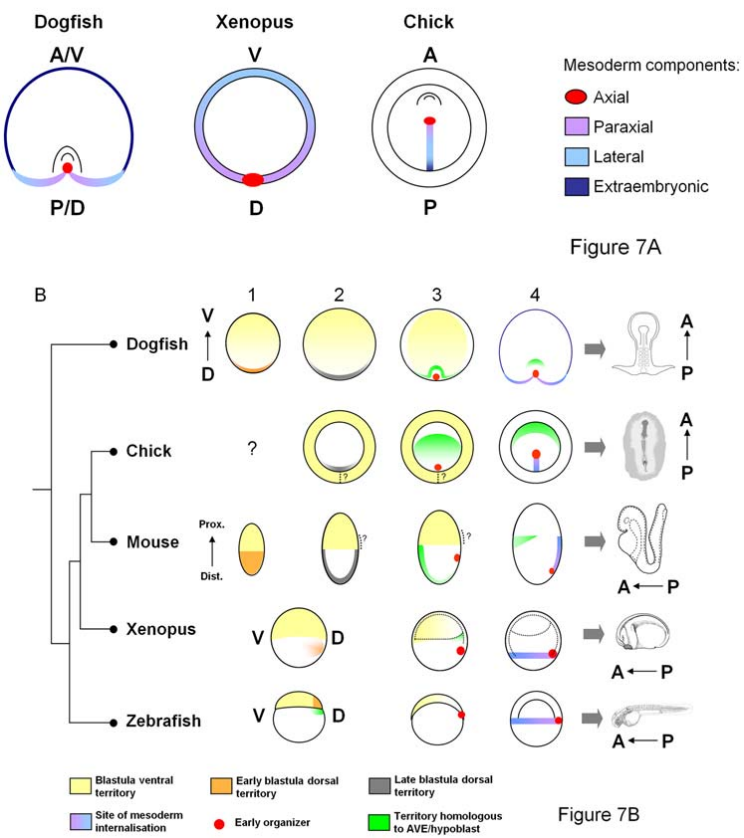
Schemes depicting territory homologies between the dogfish and vertebrate model organisms. A. Regionalisation of the site of mesoderm internalisation : comparison between the dogfish, xenopus and chick. Axial (red), paraxial (purple) and lateral (light blue) presumptive mesoderm components show the same relative location along the dogfish posterior arms, xenopus marginal ring and chick primitive streak. An additional minor mesoderm cell population, proposed to contribute to extraembryonic blood islands, is also internalised at lateral and anterior levels of the dogfish blastoderm margin (dark blue). We suggest that this cell population may be evolutionary related both to the presumptive ventral mesoderm, located to the ventral part of the marginal ring in xenopus, and extraembryonic mesoderm, internalised at the posterior part of the primitive streak in amniotes. A, anterior; P, posterior; V, ventral; D, dorsal (refer to the nomenclature paragraph in the Materials and methods section for the use of these terms in the manuscript). B. Similarities in the relative organisation of extraembryonic and embryonic territories between the dogfish and amniotes at blastula and early gastrula stages. Columns 1, 2, 3 and 4 show proposed territory homologies between the dogfish and the vertebrate model organisms at early blastula (1), late blastula (2), early gastrula (3) and mid-gastrula (4) stages respectively. A single blastula stage is shown in xenopus and zebrafish, since the conservation of the temporal sequence of gene expression inductions is less clear with these species. At early blastula stages, the dogfish blastoderm shows a partitioning into two territories, which on the basis of *Bmp4*, *Nodal* and *Otx* expressions, can be related to dorsal (orange) and ventral (yellow) territories of early zebrafish or xenopus embryos, as well as to inner cell mass (orange) and trophectoderm (yellow)-derived territories of the mouse egg cylinder. In the dogfish, this early polarity can be aligned with the later antero-posterior axis of the embryo proper (schematised on the right of the figure). At late blastula stages (column 2), homologous dorsal territories (shown in gray) expressing *Gsc*, *Lim1* and *Otx* are induced at the level of the dogfish posterior margin, chick Koller's sickle, mouse embryonic visceral endoderm. Comparisons between the three species suggest that a posterior expansion of the blastula *Bmp4* positive extraembryonic territory has taken place in amniotes (columns 1, 2, 3). Whether a remnant of the site of posterior fusion hypothesized by this evolutionary model (dotted line in columns 2, 3, 4) may persist in amniotes remains an opened question. The early organizer (red) is induced concomitantly with the anterior displacement of the hypoblast/AVE homologs (green, column 3). The site of mesoderm internalisation (column 4) is depicted as in A. Prox., proximal; Dist., distal.

Analysis of *Brachyury* expression in the dogfish had led us to point to the posterior arms as the major site of mesoderm internalisation during embryonic axis elongation [36]. This hypothesis is further refined by the study of mesoderm regional markers. Expression patterns of *FoxA2*, *Wnt8* and *Gata6* support the conclusion that the internalisation of presumptive axial, paraxial and lateral mesoderm contributing to the embryo proper is restricted to the posterior arms, excluding the lateral and anterior sides of the blastoderm, which spread by epiboly over the yolk. This conclusion is based on the striking continuity observed between the territories of these markers at specific medial to lateral levels of the posterior margin and in the newly formed embryonic axis, which is suggestive of cellular paths possibly followed by mesoderm cells towards their final destination. However, it does not preclude the possibility that a minor, extraembryonic, mesoderm cell population may also be internalised at anterior and lateral levels of the blastoderm margin, where epiboly cell movements prevail. We actually observe that these territories transiently express *Brachyury* at early stages of embryonic axis formation [36 and this study]. In addition, *MafB* and *Bmp4* expression territories at these levels precisely reflect the location where extraembryonic blood islands later differentiate in the dogfish [34]. Taken together, these data support the view that during axis elongation, the blastoderm margin is divided into two sectors, an embryonic one, restricted to the posterior arms, and an extra-embryonic one, which excludes them. The former expresses markers of axial, paraxial and lateral mesoderm, spanning largely exclusive, more and more lateral territories of the posterior margin, while the latter is only positive for markers of blood islands. This regionalisation of the margin can be easily related to the one observed along the amphibian blastopore ring and teleost blastoderm margin, dorsal and ventral in these taxa corresponding to posterior and anterior in the dogfish (see Fig. 7A). It is also clearly reminiscent of the regionalisation of the primitive streak in the chick and the mouse (Fig. 7A). Detailed fate maps in these species have thus shown a correlation between the final destination of cells in the different axial, paraxial, lateral and extraembryonic mesoderm components, and their position along the streak, from anterior to posterior levels [66]–[68]. Such a distribution exactly corresponds to the one inferred from posterior to lateral and then anterior levels of the blastoderm margin in the dogfish (Fig. 7A). As such, the broad characteristics of the dogfish gastrula are unexpectedly reminiscent of those displayed by a reptile-like amniote ancestor hypothesized by Arendt and Nübler-Jung [69] to account for the transition from an amphibian- to amniote-type gastrulation. The main difference is that a residual mesoderm cell population, likely to contribute to extraembryonic blood islands, persists in the dogfish. Taken together, these data suggest that the rise of novel adaptative modalities, such as the increase of the amount of yolk, may have independently involved convergent modifications of the gastrulation process in the chondrichthyan and amniote lineages. From a developmental standpoint, we also note that the dogfish embryo is amenable to highly straightforward interpretations, the medial to lateral distribution of presumptive mesoderm inferred from the molecular characterization along the posterior arms precisely reflecting its later location in the embryonic axis. The proposed link with amniotes therefore provides a comparative framework to understand the relatively complicated cell movements and counter-intuitive distribution of axial to lateral mesoderm progenitors along anterior to posterior level of the streak of amniotes [66].

### Evolution of forebrain induction mechanisms in jawed vertebrates

#### Hypoblast and organizer equivalents in the dogfish embryo

Cell populations homologous to the Spemann organizer have been unambiguously recognised in all vertebrate model organisms, on the basis of largely similar genetic and inductive properties [70]–[73]. A detailed molecular characterisation has also recently pointed to the presence of a homologous territory in a cephalochordate [74], suggesting an ancient origin among chordates. The conservation of the hypoblast is more difficult to assess. This extraembryonic territory, termed hypoblast in the chick and anterior visceral endoderm in the mouse, plays essential roles in head formation in amniotes [21]–[23]. While the underlying molecular signals, which involve a transient and localized repression of Nodal and Wnt signalling, appear shared by all vertebrate model organisms and even Amphioxus [27], [74]–[82], the conservation of a distinct territory homologous to the amniote hypoblast is far from clear, even in amphibians and teleosts. The xenopus deep or suprablastoporal endoderm and the zebrafish dorsal yolk syncitial layer have been proposed to correspond to homologues of the hypoblast, primarily on the basis of *Hex* or *Cerberus* expression [27]–[29], [31], [32]. However, evidence for the specific roles demonstrated in the mouse and chick is still lacking [30]. In the dogfish, the molecular characterization clearly points to the presence of two distinct expression territories, respectively located at the level of the posterior margin and the notochordal triangle, both expressing markers of the hypoblast and early organizer such as *Otx*, *Lim1*, *Gsc* and *FoxA2*. These two expression phases are clearly induced in response to different genetic cues, as shown by the analysis of the three *Otx* paralogues, expressed in these two territories in different combinations, either *Otx1* and *Otx5*, or *Otx2* and *Otx5*. The location and timing of gene expression in these two domains also point to a number of correlations, which support their respective identification as hypoblast and early organizer homologues. As the mouse embryonic visceral endoderm or chick hypoblast, the first phase of expression is thus induced prior to the onset of *Brachyury* expression and it does not overlap with its domain when gastrulation begins. It also shares with the hypoblast of amniotes a characteristic anterior displacement, initiated at pre-gastrulation stages in the lower layer, towards the site of head formation. Finally, its location and timing of expression closely correlates with an extinction of *Wnt8* signal at the posterior margin, which is consistent with the hypothesis of a repression of Wnt8 mediated posteriorising signals [83]–[86]. The second territory expressing hypoblast and early organizer markers markedly differs in several respects. In line with its identification as an organizer homologue, it becomes detectable slightly later and is restricted to a discrete, medial cell population. This expression territory also overlaps with the earliest epiblast *Brachyury* expression and is next observed in the prechordal plate or axial mesendoderm, known as organizer derivatives. Together with the molecular characterization, these characteristics point to the stage 10 posterior margin and the stage 11 notochordal triangle as hypoblast and early organizer homologues in the dogfish. This implies that the presence of a hypoblast homolog, distinct from the organizer, is a gnathostome characteristic. Unlike the zebrafish, this cell is not syncitial in the dogfish, in line with the derived character of the yolk syncitial layer in actinopterygians [87].

#### A two-step process of forebrain induction in the dogfish as in amniotes

In the chick, the induction of early anterior neuroectoderm markers involves at least two distinct specification steps, which respectively rely on signals secreted by the hypoblast and the early organizer [23], [88], [89]. The first one, which can be triggered by signals secreted by the hypoblast, corresponds to an unstable specification state and is often referred to as “pre-neural/pre-forebrain” state. The second one involves a distinct set of signals, which stabilize expression of early anterior neuroectodermal markers and emanate from the early organizer or its derivatives. In the mouse, both experimental and genetic evidence support the conservation of these two specification states [90]–[94]. In contrast, they have not been demonstrated thus far in xenopus and zebrafish [reviewed in 95]. In the dogfish, two successive phases of *Otx2* expression are observed, an initial diffuse one in the stage 10–11 epiblast and a later one in the morphologically distinct anterior neural plate at stage 12. These two territories are clearly submitted to distinct regulations, as shown by the coexpression of the paralogous gene *Otx1* in the latter but not the former territory. The progressive concentration of the signal around the site of early organizer formation, which leads to the second phase of expression, is consistent with an involvement of organizer signals in its induction, suggesting that it may correspond to the second, stabilised neuroectoderm phase described in amniotes. This process parallels the segregation of the neural plate and surface ectoderm and could thus correspond to a cell fate decision between both lineages, in line with a conserved dorsalising effect of the organizer on the ectoderm [reviewed in 96], [97]. Whether the earlier epiblast phase of *Otx2* expression could correspond to the “pre-forebrain” state described in amniotes is difficult to assess in the absence of direct experimental approaches but is supported by its timing of induction, which takes place concomitantly with the onset of hypoblast markers expression. This early *Otx2* expression territory appears largely complementary to a presumptive mesendoderm territory expressing *Nodal*, *Brachyury* and *Wnt8*, suggesting that it may be related to an ectoderm, versus posterior mesoderm, or mesendoderm, specification state, also supported by different lines of experimental evidence in model organisms [98]–[101]. Taken together, these data support the conclusion that in the dogfish the induction of anterior neuroectoderm proceeds in two steps, as proposed in the modified Nieuwkoop's model [95].

### A conserved sequence of inductions preceding mesendoderm specification at the posterior margin of the dogfish blastoderm

Genetic analyses in model organisms provide increasing evidence that the induction of mesendoderm relies on a complex regulatory cascade, involving FGF, Wnt and TGF-β related signals [reviewed in 102]. The temporal sequence of the corresponding specification steps has been best described in the chick. In this species, the coexpression of *Wnt8* and *Vg1* at the level of the posterior marginal zone corresponds to the first molecular asymmetry indicative of the site of axis formation and is required to induce expressions of both *Nodal* and *Brachyury* in the adjacent posterior epiblast [10], [103]. This induction does not immediately result in primitive streak formation, due to a transient repression of Nodal signalling by the underlying hypoblast at the level of Koller's sickle [75]. The release of this inhibition by the anterior displacement of the hypoblast leads to the formation of the primitive streak and the expression of Lefty, a Nodal feedback inhibitor [75]. In the dogfish, the close temporal succession between the transient *Vg1* and *Wnt8 co*-expression phase at the posterior margin, the marked intensification of *Nodal* expression in the presumptive mesendoderm and the onset of *Brachyury* and *Lefty* expressions at the periphery of the blastoderm is strikingly reminiscent of the sequence of genetic interactions described in the chick. Together with accumulating functional evidence in vertebrate model organisms [102], this suggests that mesendoderm induction relies on a highly conserved genetic network involving Vg1, Nodal and Lefty signalling activities. Furthermore, we note a marked, transient sharpening of the borders of *Bmp4* expression territory in the dogfish stage 11 embryo concomitant with the onset of Nodal expression in the adjacent presumptive mesendoderm. This correlation, which has not been reported thus far either in xenopus or zebrafish, is consistent with a stimulation of *Bmp4* expression by adjacent Nodal signals, as reported in the mouse [104]–[106]. The molecular characterization of the dogfish embryo therefore highlights strong similarities with amniotes in the temporal sequence of the genetic inductions that take place not only during, but also prior to gastrulation. It also suggests that these interactions, which take place between the posterior marginal zone and adjacent Koller's sickle in the chick, follow each other, with a sharp temporal regulation, at the dogfish posterior margin.

### Early polarities in the dogfish blastula: implications for the evolution of the early dorso-ventral axis

In the dogfish, the blastocoele is associated, starting from the earliest stages of its formation, to the posterior side of the blastoderm [34], where the organizer and embryonic axis later form. Expression analyses of *Bmp4*, *Nodal* and *Otx5* show that a molecular asymmetry defines the organizer side of the blastoderm prior to the first appearance of this morphological landmark. This implies that long before the induction of *Vg1* and *Wnt8* at the posterior margin and the appearance of the embryonic axis proper, the dogfish blastoderm already exhibits a bilateral symmetry, defined by opposite *Otx5*/*Nodal* and *Bmp4* expression territories. This early polarity, which reflects the later organizer-aborganizer axis, is clearly reminiscent of the early dorso-ventral axis of amphibians and teleosts [9], [107]–[109]. A link with amniotes can also be proposed. In the mouse as in the chick, *Bmp4* is expressed in a territory which exclusively contributes to extraembryonic tissues at late blastula stages [110], [111]. In the dogfish, morphological analyses suggest that *Bmp4* expression territory similarly mainly contributes to the extraembryonic ectoderm that spreads by epiboly over the yolk at late blastula stages (stages 9–10). However, while it exhibits a radial symmetry, surrounding embryonic, *Otx2* and *Nodal* positive territories in amniotes, it is restricted to the opposite, ab-organizer, side of the blastoderm in the dogfish (see Fig. 7B, compare column 2 in the dogfish, chick and mouse). Comparisons between these two modes of development suggest that amniote evolution may have involved a posterior expansion and fusion of *Bmp4* positive extraembryonic tissues, exactly as predicted by the theoretical model proposed by Arendt and Nübler-Jung [69]. A related process actually also takes place in the dogfish albeit at much later stages, the extraembryonic yolk sac expanding both laterally and posteriorly over the yolk to fuse posteriorly to the vitelline duct that connects the embryo to the yolk [34]. This evolutionary scenario implies that at blastula stages, *Bmp4* positive territories of jawed vertebrates, such as the amphibian or teleost ventral animal cap, the mouse extra-embryonic ectoderm, the chick area opaca and the dogfish ab-organizer moiety of the blastoderm, all derive from the same ancestral territory, i. e. are homologous, even though they later follow very different fates (embryonic surface ectoderm in xenopus and zebrafish, extraembryonic tissues themselves exhibiting highly diversified differentiation pathways in the dogfish, mouse and chick). Another outcome is that at blastula stages, the genetic mechanisms that pattern the embryo along the dorso-ventral (or organizer-aborganizer) axis of amphibians, teleosts and chondrichthyans, might be related to those which act along the embryonic-extraembryonic (distal-proximal in the mouse) axis of amniotes, their similarities reflecting conserved ancestral characteristics. An essential remaining question raised by this model is to know whether the radial expansion of the ancestral ventral territory is complete in amniotes whatever the stage, thus leading to a true radial symmetry, or whether a remnant of the site of posterior fusion leaves an asymmetry (see Fig. 7B). The accumulating data showing a bilateral symmetry of the blastocyst [15]–[17] and suggesting that the mouse extraembryonic ectoderm might be endowed with an early polarity [26], lend support to the latter view.

### An ancestral link between the organizer-aborganizer polarity of the blastula and the antero-posterior polarity of the embryonic axis

A striking feature of the dogfish embryo is that the early *Bmp4*-*Nodal/Otx5* axis is precisely aligned with the future head to tail axis of the embryo (see Fig. 7B). These correlations suggest that the early patterning of the dogfish blastoderm may convey a positional information controlling the rostro-caudal polarity of the embryonic axis at the earliest stages of its formation. This hypothesis is actually consistent with available functional data in model organisms, taking into account the territory homologies proposed between the dogfish and other vertebrates. In amphibians and zebrafish, a link between early dorso-ventral and later antero-posterior patterning is clear, dorsalised and ventralised phenotypes being characterized respectively by head or trunk-tail expansions at the expense of each other [112]–[115]. However, it remains difficult to know whether the very first antero-posterior asymmetries are established prior to gastrulation or whether mesoderm is required for this process. In the mouse, our evolutionary model raises the possibility of a conserved link between the embryonic-extraembryonic organisation (distal-proximal axis in the mouse, proposed here to be related to the dorso-ventral axis of amphibians or teleosts) and the later rostro-caudal patterning of the embryo proper. This idea is currently emerging from several recent analyses of mouse mutants, showing that defects along the the proximo-distal axis of the conceptus at early post-implantation stages and along the antero-posterior axis of the embryo proper at the onset of gastrulation are frequently associated [17] [25] and is also supported by the radialisation of *Otx2* expression territory observed in *Bmp4-/-* mutants (this study). The major mechanism involved may be a restriction of the extent of the anterior visceral endoderm by extraembryonic ectoderm prior to gastrulation, as directly shown by ablation experiments [24]–[26]. These data support the view that in the mouse, early genetic interactions along the proximo-distal axis control cell allocation to head versus trunk and tail territories. In the dogfish, the relative location of the proposed equivalents of the anterior visceral endoderm (stage 10 posterior margin) and extraembryonic ectoderm (*Bmp4* positive territory) at opposite poles of the blastoderm supports the possibility of homologous antagonistic interactions. It is also consistent with a conserved role of the earliest *Nodal* phase in the specification of the anterior visceral endoderm homologue in the dogfish, as demonstrated in the mouse [104], [116]. Thus, despite the extensive morphological divergence between those species, the molecular characterization of the dogfish embryo and functional analyses in model organisms can be integrated into a coherent model of early antero-posterior axis specification involving two crucial steps. In a first one, genetic mechanisms acting at blastula stages determine the relative expansion of a dorsal and a ventral territory, respectively homologous to the anterior visceral endoderm and extra-embryonic ectoderm in the mouse. In a second step, at the onset of gastrulation, these two territories in turn control cell allocation to head versus trunk-tail embryonic territories. The relative phylogenetic position of the dogfish and amniotes support the ancestrality of this two-step process in jawed vertebrates.

### Conclusion

In this work, we have used a systematic comparative approach, aimed at understanding the link between the vertebrates, as a complement to the mechanistic approaches conducted in model organisms. A crucial point has been the use of a non-model organism, chosen both for its key phylogenetic position among vertebrates and its developmental characteristics. The resulting model integrates the mechanisms of early head and antero-posterior axis specification proposed in the vertebrate model organisms in a unifying view and also leads to predictions supported by preliminary results obtained in a mouse mutant. Important issues will be to assess how the different model organisms have, or have not diverged from this general basic pattern and to gain insight into the gene or regulatory network recruitments, which account for species- or taxa-specific adaptations. It will be equally important to directly test how far the genetic mechanisms, which control the establishment of this conserved pattern, are themselves conserved across and beyond vertebrates.

## Materials and Methods

### Nomenclature

Throughout this manuscript, anterior and posterior refer to the future relative location of head versus trunk and tail territories, but not to the corresponding presumptive territories. In the last paragraph of the discussion we preferred the use of dorsal and ventral to refer respectively to the posterior/organizer and anterior/ab-organizer sides of the blastoderm, as in xenopus or zebrafish. This terminology was chosen here to avoid any confusion with the rostro-caudal (head-tail) polarity of the embryo proper.

### Dogfish embryos

Freshly laid *S. canicula* eggs were obtained from the Biological Station of Roscoff and kept at 15°C in oxygenated seawater. Embryos were dissected and staged according to [34].

### Histological description of S. canicula embryos

For hematoxylin/eosin staining, *S. canicula* embryos were fixed in paraformaldehyde 4% in PBS (PFA 4%), dehydrated in methanol, incubated in butanol overnight and embedded in paraffin for microtome sectioning at 5 µm. Staining was performed in a 5% hematoxylin/eosin solution for 1 minute. Slides were mounted in Eukitt.

For rhodamine-phalloidin staining, embryos were fixed in PFA 4% and embedded in gelatin before cryostat sectioning at 10 µm. Slides were post-fixed in PFA 4%, rinsed twice in PBS 0,1% Triton, and incubated in a solution of rhodamine-phalloidin (Molecular Probes) at 5u/ml in PBS/BSA 1% for 20 minutes. After two washes in PBS, they were mounted using Vectashield (Vector) supplemented with DAPI.

### Cloning of S. canicula probes

#### Degenerate RT-PCR

*S. canicula* probes corresponding to *Lim1*, *FoxA2*, *Bmp4*, *Gsc*, *MafB, Wnt8* and *Lefty* were amplified by RT-PCR starting from embryonic cDNA (stages 9–15), using the degenerate primers listed in Table S1 (supplementary material). The resulting fragments were subcloned in a SmaI-digested PTZ19R vector. The *S. canicula T* probe was described in [36]. The *S. canicula Otx1, Otx2* and *Otx5* probes were described in [117].

#### EST sequencing

*S. canicula Gata6*, *HoxB1*, *Cdx2*, *Mox1*, *Vg1* and *Nodal* probes were obtained from a large-scale project of cDNA sequencing currently underway. An embryonic cDNA library was constructed in the pSPORT1 vector using the Superscript plasmid system with Gateway technology (Invitrogen), starting from 4 µg mRNA of stage 9–15 dogfish embryos. The library was plated, arrayed robotically and bacterial clones were 5′ end-sequenced by the Genoscope using ABI3730xl instruments and standard protocols.

#### Sequence analysis

For each marker used in this study, the clone identity was systematically first assessed by a BLASTN search against Genbank and confirmed by a phylogenetic analysis (data not shown). For the latter, homologous chordate sequences were retrieved using a TBLASTN search [118] against the non-redundant database at NCBI. The corresponding sequences were included into an alignment, which was used to construct a Neighbor Joining phylogenetic tree using MEGA version 3.1 [119].

### Analysis of mouse Bmp4 null-mutant embryos

Mice from line B6.129S2-Bmp4^tm1Blh^/J were obtained by courtesy of Dr Benoit Robert and are described in [120]. *Mus musculus Dkk1* and *Otx2* probes were amplified by RT-PCR starting from mouse embryonic cDNA (stage 6.5–7.5 dpc) using respectively the following primers pairs: TCTATGAGGGCGGGACA/ATTGCTGGCTTGATGGTGA and ATGATGTCTTATCTAAAG/TCACAAAACCTGGAATTT. PCR products were subcloned in a EcoRI-HindIII or EcoRI-BamHI digested PTZ19R vector.

### Whole mount in situ hybrization of dogfish and mouse embryos

Digoxigenin-11-UTP (Roche) labelled antisens RNA probes for *S. canicula* *Otx1*, *Otx2*, *Otx5*, *Lim1*, *FoxA2*, *Gsc, MafB, Lefty* and Mus musculus *Dkk1* and *Otx2*, were synthesized from linearized plasmid templates using T7 RNA polymerase. For the markers retrieved from the cDNA library, a PCR template was obtained starting from a plasmid DNA minipreparation, using the following primers: AAAGCTGGTACGCCTGCA and TAATACGACTCACTATAGGGAGAG CGTACGTAAGCTTGGATC, which leads to the addition of a T7 promoter (underlined) in antisense orientation.

Whole mount in situ hybridizations were performed according to standard procedures. For double in situ hybridization, one probe was labeled with Digoxigenin-11-UTP and the other with Fluorescein-12-UTP (Roche). RNA were detected sequentially using alkaline-phosphatase-coupled anti-DIG antibody (Roche) with BM purple (Roche) as a substrate, and HRP-coupled anti-Fluorescein antibody (Roche) with DAB (Vector) as a substrate. For histological analysis following whole-mount hybridizations, embryos were embedded in paraffin before microtome sectionning at 10 µm. Slides were counterstained with eosin (2%).

### Xenopus embryos and microinjections

*Xenopus* eggs were obtained from females injected with 500 IU of human chorionic gonadotropin (Sigma), and artificially fertilized. Eggs were dejellied with 2% cysteine hydrochloride (pH 7,8) and embryos were staged according to [121].

Capped mRNAs were synthesized from linearized plasmids using SP6 RNA polymerase (Roche) in the presence of 500 µM 5′-mGpppG-3′ cap analog, 500 µM each rUTP, rATP, rCTP and 50 µM rGTP. Synthetic mRNA was purified using a Sephadex G-50 column (Pharmacia). Microinjection of embryos was performed in 0.1× Modified Barth's Solution (MBS) containing 3% Ficoll 400. They were maintained in this solution for three hours and then cultured in 0.1×MBS to appropriate stages.

## Supporting Information

Table S1 Primers used to amplify dogfish genes
(0.03 MB DOC)
