# Supplementary material for: Evolution of Axis Specification Mechanisms in Jawed Vertebrates: Insights from a Chondrichthyan
Source: PLoS One. 2007 Apr 18;2(4):e374. doi: 10.1371/journal.pone.0000374 (PMC1847705; doi:10.1371/journal.pone.0000374)
Supplement: Table S1 — Primers used to amplify dogfish genes (0.03 MB DOC) [file pone.0000374.s001.doc]

| ***probe*** | ***Primer forward*** | ***Primer reverse*** | ***probe length*** | ***Accession number*** |
| --- | --- | --- | --- | --- |
| ***Lim1*** | TGYAARAAYGAYTTYTT (CKNDFF) | GGNCCYTGNGGRAARAA (FFPQGP) | 711 bp | AY217780 |
| ***FoxA2*** | ATHACNATGGCNATHCA (ITMAIQ) | TGYTCNGGYTTNARRTG (HLKPEH) | 537 bp | AY217779 |
| ***Lefty*** | TTYGAYGTNACNCARGC (FDVQTA) | ATCCARTAYTGNGTCCA (WTQYWI) | 303 bp | EF174301 |
| ***MafB*** | ATGAAGTTYGARGTNAAG (MKFEVK) | KCCRCGRTTCTTNARNGT (TLKNRG) | 615 bp | EF174302 |
| ***Gsc*** | GGNATGTTYWSNATHGA (GMFSID) | CCNACRTCNGGRTAYTT (KYPDVG) | 492 bp | EF174299 |
| ***Bmp4*** | ATHAAYATHGARTAYGT (INIEYV) | TTNACNAYNGTRTGNACDAT (IVQTLVN) | 495 bp | EF174300 |
| ***Wnt8*** | GGN ATH GAR GAR TGY AA (GIEECK ) | CA YTC NCK NCC YTC NGT (TEGREC) | 657 bp | EF174298 |

**Supplementary Material: Primers used to amplify dogfish genes**
